# The Gut Microbiota Dysbiosis in Preeclampsia Contributed to Trophoblast Cell Proliferation, Invasion, and Migration via lncRNA BC030099/NF-*κ*B Pathway

**DOI:** 10.1155/2022/6367264

**Published:** 2022-06-24

**Authors:** Rong Tang, Gong Xiao, Yu Jian, Qiongjing Yuan, Chun Jiang, Wei Wang

**Affiliations:** ^1^Department of Nephrology, Xiangya Hospital, Central South University, Changsha, 410078 Hunan, China; ^2^Department of Obstetrics and Gynecology, Xiangya Hospital, Central South University, Changsha, 410078 Hunan, China

## Abstract

**Background:**

Preeclampsia (PE) is the main reason of maternal and perinatal morbidity and mortality. Gut microbiota imbalance in PE patients is accompanied by elevated serum lipopolysaccharide (LPS) levels, but whether it affects the occurrence and development of PE, the underlying mechanism is not clear. This paper intends to investigate the relationship between lncRNA BC030099, inflammation, and gut microbiota in PE.

**Methods:**

The feces of the patients were collected, and gut microbiota changes were assessed by 16S rRNA sequencing and pathway analysis by PICRUSt. Next, we examined LPS and lncRNA BC030099 levels in feces or placenta of PE patients. Then, we knocked down lncRNA BC030099 in HTR-8/SVneo cells and added the NF-*κ*B pathway inhibitor JSH-23. CCK-8 and Transwell assays were performed to determine cell proliferation, migration, and invasion. Western blot was utilized to evaluate MMP2, MMP9, snail, and E-cadherin, p-I*κ*B*α*, I*κ*B*α*, and nuclear NF-*κ*B p65 levels. IL-6, IL-1*β*, and TNF-*α* levels were examined by ELISA.

**Results:**

Gut microbiota was altered in PE patients, and microbial genes associated with LPS biosynthesis were significantly elevated in gut microbiota in the PE group. LPS level in feces and placenta of PE group was significantly elevated. lncRNA BC030099 level in placenta of PE group was also notably promoted. Knockdown of lncRNA BC030099 promoted HTR-8/SVneo cell proliferation, migration, and invasion. Knockdown of lncRNA BC030099 also elevated MMP2, MMP9, and snail levels and repressed E-cadherin level. In addition, lncRNA BC030099 affected NF-*κ*B pathway. Furthermore, NF-*κ*B inhibitor reversed HTR-8/SVneo cell proliferation, invasion, and migration induced by LPS.

**Conclusions:**

The gut microbiota dysbiosis in PE contributed to HTR-8/SVneo cell proliferation, invasion, and migration via lncRNA BC030099/NF-*κ*B pathway.

## 1. Introduction

Preeclampsia (PE) is a hypertensive disorder of pregnancy involved in 2% to 8% of pregnancy-related complications worldwide [1]. It is the main reason of maternal and perinatal morbidity and mortality [2]. The etiology and pathogenesis of PE have not been fully clarified. Currently, it is mainly believed to be related to insufficient remodeling of uterine spiral arterioles, excessive activation of inflammatory immunity, and damage to vascular endothelial cells [3]. The clinical treatment measures for PE are limited, focusing on the control of acute hypertension, prevention of PE, and timely delivery, and the only effective treatment is delivery of the fetus and placenta [4]. Therefore, an in-depth understanding of the pathogenesis of PE can provide guidance for clinical prediction and prevention of PE, avoid serious complications in pregnant women, and improve pregnancy outcomes.

Long noncoding RNAs (lncRNAs) are a class of RNA molecules with transcripts > 200 nt, which can regulate target gene expression via epigenetic, transcriptional, and posttranscriptional regulation. It is involved in different biological processes including cell differentiation and proliferation, apoptosis, and necrosis [5, 6]. In addition, abnormal lncRNA expression is associated with disease progress [7]. Therefore, lncRNAs' role in PE pathogenesis has also attracted much attention. Luo et al. screened lncRNA expression profiles in PE patients' placentas, suggesting abnormal lncRNA expression may be one of the causes of PE [8]. Based on this, Sun et al. detected 5 known uterus-related lncRNAs' expression in 48 PE patients and 24 non-PE healthy subjects and found lncRNA BC030099 expression was significantly facilitated, indicating elevated plasma level of lncRNA BC030099 is related to an accelerated risk of PE and can be used as the potential biomarker to predict the occurrence of PE [9], but whether it is involved in the disease progression of PE and its specific mechanism still need to be further explored.

Gut microbiota is a type of microbial population that inhabits the human gut, with a large number and variety, participating in the metabolism of the human body and maintaining the homeostasis of the human body [10]. Recently, the relationship between gut microbiota changes and pregnancy diseases has received extensive attention. Studies have found that immune tolerance, inflammatory response, abnormal glucose, and lipid metabolism, and oxidative stress caused by gut microbiota imbalance may be involved in PE pathogenesis [11, 12]. The study of gut microbiota provides new targets and ideas for preventing and treating PE. Lipopolysaccharide (LPS), as a product of gut microbiota, can cause inflammation in body and is associated with various disease occurrence and development. Studies have revealed gut microbiota imbalance in PE patients is accompanied by increased serum LPS level [12], but whether it affects the occurrence and development of PE, the underlying mechanism is not clear. Furthermore, accumulative evidence suggests that lncRNAs could affect LPS-induced PE animal or cell models through various signaling pathways. Huang et al. constructed PE rat models induced by LPS and revealed that overexpression of lncRNA Uc.187 could induce PE-like symptoms in a pregnant rat model by affecting the distribution of *β*-catenin in the cytoplasm and nucleus [13]. Chen et al. used LPS to treat HTR-8/SVneo cells and found that lncRNA KCNQ1OT1 could target the regulation of miR-146a-3p through the CXCL12/CXCR4 pathway in the proliferation, invasion, and migration of HTR-8/SVneo cells [14]. However, the mechanism of lncRNA BC030099 on PE is unclear.

Therefore, based on the above background, this research intends to explore lncRNA BC030099, inflammation, and gut microbiota relationship in PE. Our research may provide new targets for diagnosing and treating PE, as well as new ideas for the study of the pathological mechanism of PE.

## 2. Materials and Methods

### 2.1. Collection of Clinical Samples

We collected feces and placental tissue from PE patients in Xiangya Hospital central South University as the PE group (*n* = 6). The healthy subjects during the same period were selected as the control group (C group, *n* = 8). The inclusion criteria of this study were as follows: aged at 25-40 years; gestational weeks from 32 to 39; singleton; no fetal abortion and stillbirth; no history of adverse pregnancy and childbirth; and no history of assisted reproduction; no history of smoking, drinking, and other drugs. The exclusion criteria were multiple pregnancy; diabetes, chronic hypertension, and kidney disease or other pregnancy complications before pregnancy; antibiotics, glucocorticoids, and immunosuppressants within 1 month before stool collection and other drugs [12]. Fecal samples were collected in stool collection tubes and stored at -80°C until further processing.

### 2.2. 16S rRNA Sequencing and PICRUSt Pathway Analysis

Fecal samples from healthy subjects (*n* = 8) and PE patients (*n* = 6) were collected to detect changes in microbial diversity. Illumina NovaSeq PE250 was applied for 16S amplicon sequencing to obtain raw data. Sequence data analysis mainly used Qiime 2 (Qiime2-2020.2) and R software (4.0.2). In addition, KEGG pathway analysis was performed by PICRUSt.

### 2.3. Enzyme Linked Immunosorbent Assay (ELISA)

According to LPS (CSB-E09945h, CUSABIO, China), IL-1*β* (CSB-E08053h, CUSABIO, China), TNF-*α* (CSB-E04740h, CUSABIO, China), and IL-6 (CSB-E04638h, CUSABIO, China) ELISA instructions, we detected LPS level in feces and placental tissues and IL-6, IL-1*β*, and TNF-*α* levels in cells. 20 mg of feces and placental tissue was taken, respectively, and the blood stains were washed with 1 × PBS. Feces and placental tissue were cut into small pieces and put into tissue grinder (homogenate tube), 200 *μ*L 1 × PBS was added to make homogenate, then put in -20°C overnight. After repeated freeze-thaw treatment two times to destroy the cell membrane, the tissue homogenate was centrifuged at 5000 g (2-8°C) for 5 min to take the supernatant, which should be detected. For cells, centrifugation was performed at 1000 g (2-8°C) for 15 min, and the supernatant should be immediately detected.

### 2.4. Cell Treatment

Human trophoblast cell HTR-8/SVneo was purchased from Tongpai (Shanghai, China) Biotechnology Co., Ltd., and culture conditions were as follows: RPMI-1640 medium and 10% fetal bovine serum (FBS). LPS was applied to induce cellular inflammation [15], and in addition, lncRNA BC030099 was knocked down, which are grouped into the si-control, si-con+LPS, si-BC030099, and si-BC030099+LPS groups. In addition, we added 10 *μ*M NF-*κ*B-specific inhibitor JSH-23 (Merck, Germany) [16], grouped as the control, LPS, JSH-23, and LPS+JSH-23 groups. lncRNA BC030099 expression was knocked down. si-BC030099 and negative control si-control were synthesized by Sangon Biotechnology Co., Ltd. (Shanghai, China).

### 2.5. Quantitative Real-Time PCR (qRT-PCR)

Total RNA was extracted using Trizol method, RNA was reverse transcribed into cDNAs using a cDNA reverse transcription kit (#CW2569, Beijing ComWin Biotech, China), and fluorescence quantification was applied by Ultra SYBR Mixture (#CW2601, Beijing ComWin Biotech, China). The relative expression of genes was examined on an PCR instrument (QuantStudio1, Thermo, USA). Using GAPDH as an internal reference, the 2^-*ΔΔ*Ct^ method was performed to calculate lncRNA BC030099 level. The primers were as follows: lncRNA BC030099-F: GCCTCCATCCTTTCAGACCC and lncRNA BC030099-R: GCCCTTGGAAAGTGTCAGGA; GAPDH-F: ACAGCCTCAAGATCATCAGC and GAPDH-R: GGTCATGAGTCCTTCCACGAT.

### 2.6. Western Blot

Total protein was extracted from cells by RIPA lysis buffer (P0013B, Beyotime, China), followed by protein quantification, mixed with SDS-PAGE loading buffer, and protein was adsorbed on PVDF membrane by gel electrophoresis. MMP2 (10373-2-AP, 1 : 500, Proteintech, USA), MMP9 (ab76003, 1 : 5000, Abcam, UK), snail (13099-1-AP, 1 : 500, Proteintech, USA), and E-cadherin (20874-1-AP, 1 : 1000, Proteintech, USA), I*κ*B*α* (ab32518, 1 : 500, Abcam, UK), p-I*κ*B*α* (ab133462, 1 : 10000, Abcam, UK), and nuclear NF-*κ*B p65 (10745-1-AP, 1 : 1000, Proteintech, USA) primary antibodies and *β*-actin (66009-1-Ig, 1 : 1000, Proteintech, USA) were incubated overnight at 4°C. HRP secondary antibodies were then incubated. Visualization was performed using ECL luminescent fluid (advansta, K-12045-D50, USA). *β*-actin was used as an internal reference to examine protein levels.

### 2.7. Cell Counting Kit-8 (CCK-8) Assay

Cells grouped above were digested and counted and seeded in a 96-well plate (5 × 10^3^ cells/well), with 100 *μ*L per well. After culturing the adherent cells, we followed the above method for the corresponding time and then added 10 *μ*L/well of CCK-8 to each well. After incubation at 37°C and 5% CO_2_ for 4 h, the absorbance (450 nm) was analyzed on the Bio-Tek microplate reader (MB-530, HEAES, China).

### 2.8. Transwell Assays

1 × 10^6^/mL cells were resuspended in serum-free medium, 100 *μ*L cell suspension was added into Transwell chamber (#33318035, Corning, USA) upper, and 10% FBS was added into lower chamber and cultured for 48 h. Chamber culture medium was discarded. Cells on upper ventricle were wiped with the wet cotton swab. 4% paraformaldehyde was fixed. Cells were stained with 0.5% crystal violet and eluted with water. Cells on the outer surface of upper chamber were observed under a microscope (Olympus, Japan) and photographed. Detection of cell invasion was performed using Transwell chamber (3428, Corning, USA) with Matrigel Basement Membrane Matrix (354262, BD Biocoat, USA). The other steps were as described above.

### 2.9. Statistical Analysis

Statistical analysis was performed using Graphpad Prism8.0 statistical software. Measurement data are expressed as the mean ± standard deviation. An unpaired *t*-test was used between groups, and one-way analysis of variance was used for comparison among multiple groups. The correlation of LPS and lncRNA BC030099 in PE placental tissues was calculated by Spearman's correlation analysis. *P* < 0.05 indicated a statistically significant difference.

## 3. Results

### 3.1. Gut Microbiota in PE Patients Was Changed, and Key Pathway Targets (LPS) Were Screened

**Figure 1:**
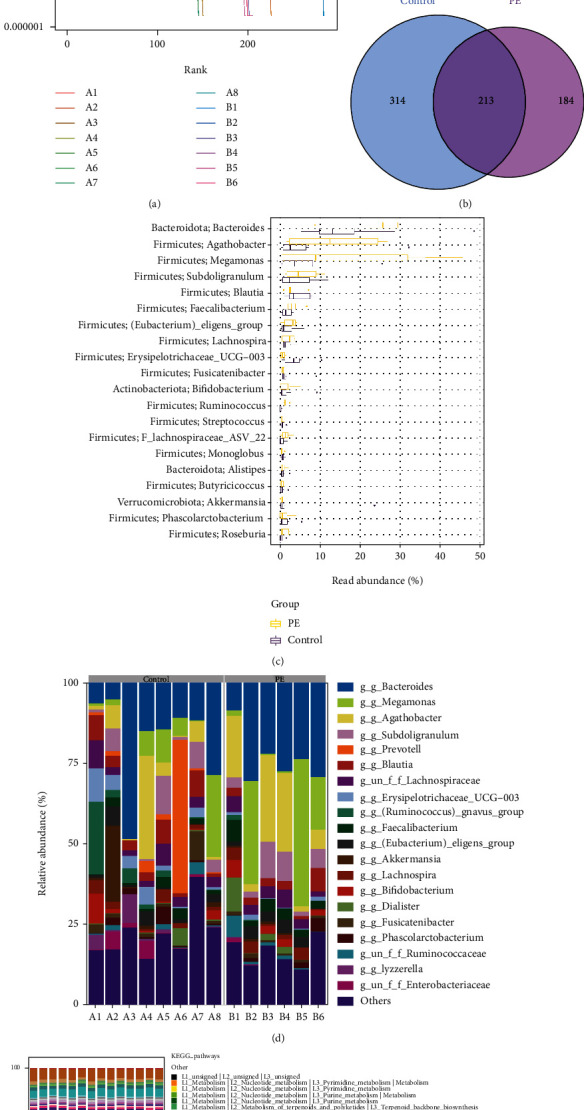
Gut microbiota in PE patients were changed, and key pathway targets (LPS) were screened. (a) Rank abundance curve. (b) Venn diagram. (c) Relative abundance histogram of top 20 ASVs at the genus level. (d) Top 20 dominant gut microbiota with relative abundance at the phylum level. (e) PICRUSt analysis in the KEGG pathway. A: control group; B: PE group.

First, we analyzed changes in gut microbiota by 16S rRNA sequencing and pathway analysis by PICRUSt.  Figure 1(a) shows the rank abundance curve. Venn diagram showed shared and unique ASVs between groups (Figure 1(b)). Among them, there were 314 ASVs unique to the control group, 184 ASVs unique to PE patients, and 213 ASVs shared by two groups. Relative abundance histogram of the top20 ASVs at genus level further suggested the abundance and composition of microbial communities in each group were different (Figure 1(c)). As shown in Figure 1(d), the relative abundance of the top 20 dominant gut microbiota at phylum level was shown. Furthermore, KEGG showed that microbial genes related to LPS biosynthesis were significantly elevated in gut microbiota in PE group (Figure 1(e)).

### 3.2. LPS Was Positively Correlated with lncRNA BC030099

**Figure 2:**
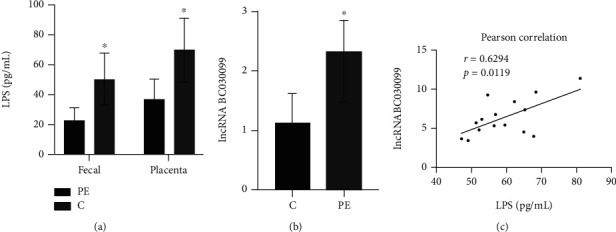
LPS was positively correlated with lncRNA BC030099. (a) LPS level in fecal and placental tissues in PE and control groups. (b) lncRNA BC030099 level in placental tissues in PE and control groups. (c) The correlation of LPS and lncRNA BC030099 in PE placental tissues were evaluated by Spearman's correlation analysis. ^∗^*P* < 0.05 vs. C (control).

Next, we examined LPS level in feces and placenta of PE patients. LPS level in the feces and placenta of PE group were significantly elevated than control group (Figure 2(a)). Furthermore, we used qRT-PCR to evaluate lncRNA BC030099 level in PE patients' placenta. lncRNA BC030099 level in placenta of the PE group was also strikingly elevated than that of the control group (Figure 2(b)). Spearman's correlation analysis showed there was a significant positive correlation between LPS and lncRNA BC030099 expression in placental tissue (Figure 2(c)).

### 3.3. Knockdown of lncRNA BC030099 Promoted Trophoblast Cell Proliferation, Invasion, and Migration

**Figure 3:**
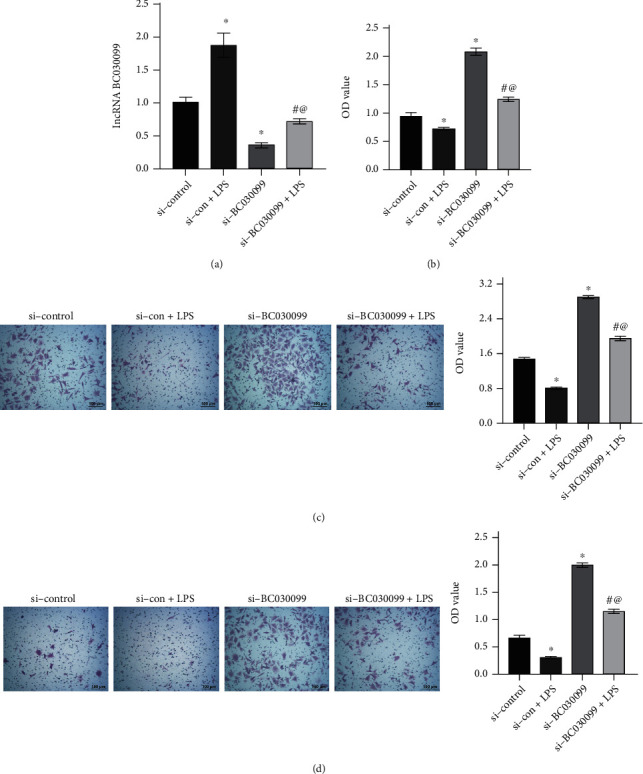
Knockdown of lncRNA BC030099 promoted trophoblast cell proliferation, invasion, and migration. (a) lncRNA BC030099 expression in trophoblast cells. (b) CCK-8 assay was utilized to measure HTR-8/SVneo cell proliferation. (c, d) Transwell assays were performed to monitor HTR-8/SVneo cell migration and invasion. Scale bar = 100 *μ*m; ^∗^*P* < 0.05 vs. si-control, ^#^*P* < 0.05 vs. si-con+LPS, and ^@^*P* < 0.05 vs. si-BC030099.

Next, we knocked down lncRNA BC030099. We found lncRNA BC030099 expression was repressed in the si-BC030099 group compared with the si-control group. This indicated that we successfully knocked down lncRNA BC030099. Compared with the si-BC030099 group, lncRNA BC030099 expression in the si-BC030099+LPS group was facilitated (Figure 3(a)). Cell function experiments revealed compared with the si-control group; the si-BC030099 group had enhanced cell proliferation, migration, and invasion abilities. However, the si-BC030099+LPS group showed reduced cell proliferation, migration, and invasion than the si-BC030099 group (Figures 3(b)–3(d)). The above results indicated knockdown of lncRNA BC030099 promoted trophoblast cell proliferation, invasion, and migration.

### 3.4. Knockdown of lncRNA BC030099 Elevated MMP2, MMP9, and Snail Levels and Repressed E-Cadherin Level

**Figure 4:**
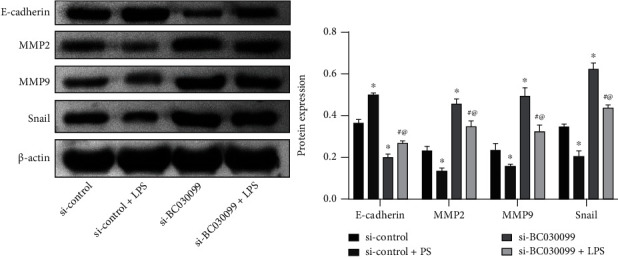
Knockdown of lncRNA BC030099 elevated MMP2, MMP9, and snail levels and repressed E-cadherin level. Western blot was utilized to assess MMP2, MMP9, snail, and E-cadherin levels in HTR-8/SVneo cells. ^∗^*P* < 0.05 vs. si-control, ^#^*P* < 0.05 vs. si-con+LPS, and ^@^*P* < 0.05 vs. si-BC030099.

In addition, Western blot analysis showed that MMP2, MMP9, and snail expressions were increased in the si-BC030099 group compared with the si-control group. But the si-BC030099+LPS group showed decreased expression of MMP2, MMP9, and snail than the si-BC030099 group. The trend of E-cadherin was opposite to that of snail (Figure 4). Collectively, knockdown of lncRNA BC030099 elevated MMP2, MMP9, and snail levels and repressed E-cadherin level.

### 3.5. lncRNA BC030099 Affected NF-*κ*B Pathway

**Figure 5:**
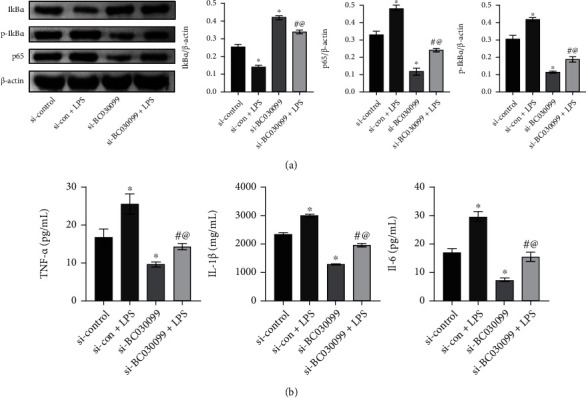
lncRNA BC030099 affected NF-*κ*B pathway. (a) Western blot was performed to determine p-I*κ*B*α*, I*κ*B*α*, and nuclear NF-*κ*B p65 levels in HTR-8/SVneo cells. (b) IL-6, IL-1*β*, and TNF-*α* levels were determined by ELISA. ^∗^*P* < 0.05 vs. si-control, ^#^*P* < 0.05 vs. si-con+LPS, and ^@^*P* < 0.05 vs. si-BC030099.

Next, we examined NF-*κ*B pathway-related protein expression. Compared with the si-control group, the si-con+LPS group showed a decrease in I*κ*B*α* expression and an increase in p-I*κ*B*α* and p65 expressions; I*κ*B*α* expression in the si-BC030099 group was promoted, and p-I*κ*B*α* and p65 expressions were inhibited. However, the si-BC030099+LPS group had repressed I*κ*B*α* expression and facilitated p-I*κ*B*α* and p65 expressions than the si-BC030099 group (Figure 5(a)). In addition, we examined inflammation-related marker expression. Compared with those in the si-control group, IL-6, IL-1*β*, and TNF-*α* levels in the si-con+LPS group were facilitated; IL-6, IL-1*β*, and TNF-*α* levels in the si-BC030099 group were repressed. The si-BC030099+LPS group had elevated IL-6, IL-1*β*, and TNF-*α* levels than the si-BC030099 group (Figure 5(b)). All in all, lncRNA BC030099 affected NF-*κ*B pathway.

### 3.6. NF-*κ*B Inhibitor Reversed Trophoblast Cell Proliferation, Invasion, and Migration Induced by LPS

**Figure 6:**
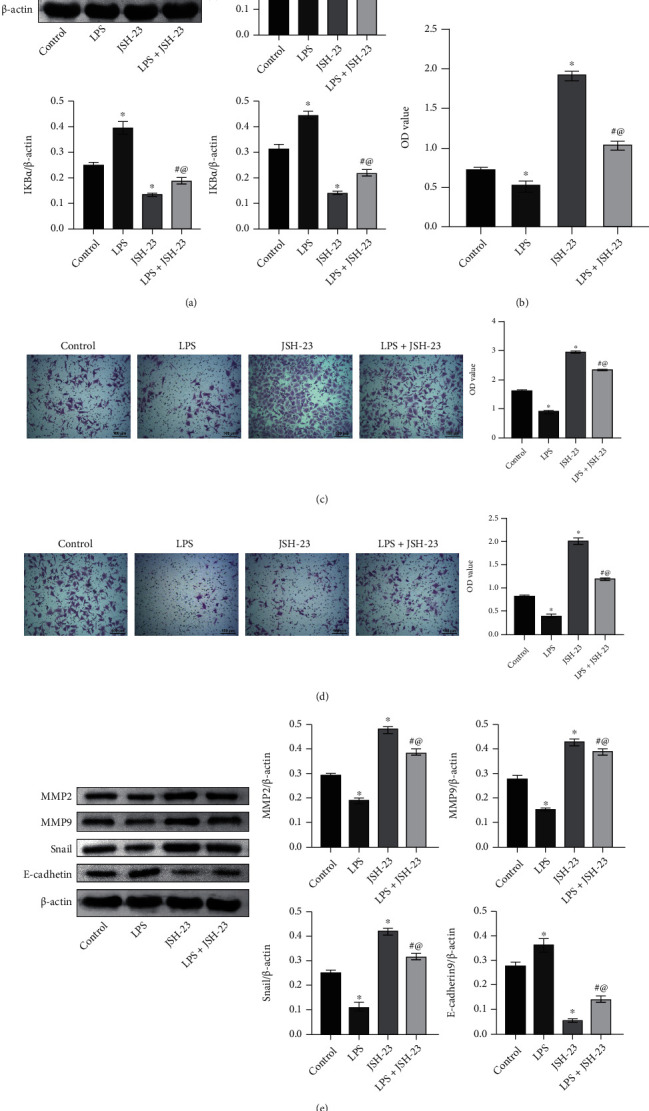
NF-*κ*B inhibitor reversed trophoblast cell proliferation, invasion, and migration induced by LPS. (a) I*κ*B*α*, p-I*κ*B*α*, and nucleus NF-*κ*B p65 expressions in trophoblast cells. (b) Cell activity was determined by CCK-8 assay. (c, d) Cell migration and invasion were examined by Transwell assays. (e) Western blot was performed to determine MMP2, MMP9, snail, and E-cadherin levels in trophoblast cells. Scale bar = 100 *μ*m. ^∗^*P* < 0.05 vs. control; ^#^*P* < 0.05 vs. LPS.

Finally, we used NF-*κ*B pathway inhibitor JSH-23. I*κ*B*α* expression was suppressed, and p-I*κ*B*α* and p65 expressions were facilitated in LPS group than in the control group. However, compared with the LPS group, the LPS+JSH-23 group showed an increase in I*κ*B*α* expression and a decrease in p-I*κ*B*α* and p65 expressions (Figure 6(a)). Cell function experiments showed compared with the control group; the LPS group had reduced cell proliferation, migration, and invasion abilities. After adding JSH-23, the LPS+JSH-23 group had enhanced cell proliferation, migration, and invasion abilities (Figures 6(b)–6(d)). Then, we used Western blot to detect MMP2, MMP9, snail, and E-cadherin levels. MMP2, MMP9, and snail expressions were suppressed in the LPS group than the control group. After adding JSH-23, MMP2, MMP9, and snail expressions were also repressed in the LPS+JSH-23 group. The trend of E-cadherin was opposite to that of snail (Figure 6(e)). The above results indicated that NF-*κ*B inhibitor reversed trophoblast cell proliferation, invasion, and migration induced by LPS.

## 4. Discussion

PE is a devastating medical complication of pregnancy that causes severe maternal and fetal morbidity and mortality [17]. PE complicates maternal and child health management and contributes to the majority of adverse pregnancy outcomes, but the mechanisms underlying PE development remain unclear. Studies have shown gut microbiota structure of PE patients has changed significantly, which may be associated with the occurrence and development of disease [18]. However, further studies are needed to understand underlying mechanisms. In this research, we sought to explore lncRNA BC030099, inflammation, and gut microbiota relationship in PE. Our research may contribute to the early diagnosis and targeted monitoring of PE.

Colonization of neonatal gut with beneficial bacteria is essential for mucosal barrier's establishment and maintenance, thereby protecting neonate from intestinal pathogens and local and systemic inflammation [19]. Recent advances show abnormalities in microbiome composition may play a role in various disease pathogenesis including PE [20]. Alterations in gut microbiota composition could alter short-chain fatty acid profile released by bacteria and contribute to hypertension and metabolic syndrome [21]. Chen et al. found that PE patients had repressed bacterial diversity and marked dysbiosis [22]. Our study is consistent with previous studies showing changes in gut microbiota in PE patients. It was reported maternal blood IL-6 levels in PE were positively correlated with Bilophila and Oribacterium, while LPS levels were negatively correlated with Akkermansia [23]. Chang et al. found the abundance of LPS synthesis pathway was significantly elevated in predicted PE patients, while the abundance of the G protein-coupled receptor pathway was observably repressed [11]. We also found that gut microbiota related to LPS biosynthesis were significantly elevated in gut microbiota in the PE group.

lncRNAs are noncoding transcripts, typically over 200 nt in length, that have recently emerged as one of the largest and significantly diverse RNA families [24]. lncRNAs have now become important players in almost all gene functions and regulatory levels [25]. The study showed circulating lncRNA BC030099 level in plasma of PE patients was significantly higher than non-preeclamptic healthy subjects. Elevated plasma lncRNA BC030099 level was related to an elevated risk of PE and may be considered a novel biomarker [9]. Through validation, we found lncRNA BC030099 level in placenta of the PE group was also markedly elevated. Furthermore, the effects of gut microbiota on host lipid metabolism may be mediated via gut microbiota-produced metabolites including short-chain fatty acids, secondary bile acids, and trimethylamine, as well as proinflammatory bacterial-derived factor LPS [26]. Wang et al.'s studies have shown that patients with PE have gut microbiota imbalance and elevated plasma LPS level [12]. We found LPS levels in the feces and placenta of PE group were significantly facilitated and LPS and lncRNA BC030099 expressions were notably positively correlated. This is the first time we report LPS and lncRNA BC030099 role in PE.

lncRNAs have been shown to participate in different biological processes including cell growth, antiapoptosis, migration, and invasion [27]. Chen et al. reported lncRNA KCNQ1OT1 can regulate miR-146a-3p role in HTR8/SVneo cell proliferation, invasion, and migration through the CXCL12/CXCR4 pathway, which may be involved in PE pathogenesis [14]. Our study also showed knockdown of lncRNA BC030099 promoted HTR-8/SVneo cell proliferation, migration, and invasion. Studies have shown that MMPs are vital mediators of vascular and uterine remodeling and reduced MMP2 and MMP9 expressions is thought to be involved in hypertensive pregnancy and PE [28, 29]. Placental extravillous cell invasion involves cell EMT, and several EMT regulators (including snail and E-cadherin) have been found to play vital roles in PE development [30]. Knockdown of lncRNA CRNDE has been reported to inhibit the proliferation, migration, and invasion of HTR-8/SVneo cells, inhibit the formation of EMT, and reduce the protein expression of MMP2 and MMP9 [31]. Zhou et al. reported that lncRNA SNHG12 promoted trophoblast cell migration and invasion by inducing the progression of EMT [32]. Consistent with their study, we found knockdown of lncRNA BC030099 elevated MMP2, MMP9, and snail levels and repressed E-cadherin level.

NF-*κ*B is a vital transcription factor for inflammation-related protein expression [33]. The NF-*κ*B p65 pathway is associated with PE, and inhibition of NF-*κ*B p65 pathway can ameliorate PE [34]. NF-*κ*B and proinflammatory cytokines have been reported to be associated with trophoblast dysfunction [35], but the underlying mechanisms remain unclear. Vaughan and Walsh reported that placental NF-*κ*B was activated nearly 10-fold in PE. Oxidative stress leads to NF-*κ*B activation in trophoblast-like cells, which is enhanced by TNF-*α* [36]. We found through cellular experiments that LPS induced the nucleus transition of NF-*κ*B through lncRNA BC030099. Furthermore, NF-*κ*B inhibitor reversed trophoblast cell proliferation, invasion, and migration induced by LPS. This is also the first time that we report the mechanism of action of LPS, lncRNA BC030099 and NF-*κ*B p65 in PE.

In conclusion, we explored the evolution of LPS, lncRNA BC030099, and NF-*κ*B p65 involved in PE progression and its mechanism. We report for the first time that gut microbiota dysbiosis in PE contributed to trophoblast cell proliferation, invasion, and migration through the lncRNA BC030099/NF-*κ*B pathway. This study provides a reference for exploring lncRNA and gut microbiota mechanism in PE.

## Data Availability

The data used to support the findings of this study are available from the corresponding author upon request.

## References

[1] Karrar S., Hong P. L. (2022). Preeclampsia. *StatPearls*.

[2] Chaemsaithong P., Sahota D. S., Poon L. C. (2022). First trimester preeclampsia screening and prediction. *American journal of obstetrics and gynecology*.

[3] Jung E., Romero R., Yeo L. (2022). The etiology of preeclampsia. *American journal of obstetrics and gynecology*.

[4] Chappell L. C., Cluver C. A., Kingdom J., Tong S. (2021). Pre-eclampsia. *Lancet*.

[5] Chen L., Wang J., Li J. W., Zhao X. W., Tian L. F. (2020). LncRNA MEG3 inhibits proliferation and promotes apoptosis of osteosarcoma cells through regulating Notch signaling pathway. *European Review for Medical and Pharmacological Sciences*.

[6] Dykes I. M., Emanueli C. (2017). Transcriptional and post-transcriptional gene regulation by long non-coding RNA. *Genomics, proteomics & bioinformatics*.

[7] Zhang Q., Wang Z., Cheng X., Wu H. (2021). lncRNA DANCR promotes the migration an invasion and of trophoblast cells through microRNA-214-5p in preeclampsia. *Bioengineered*.

[8] Xiucui Luo X. L. (2018). Long non-coding RNAs serve as diagnostic biomarkers of preeclampsia and modulate migration and invasiveness of trophoblast cells. *Medical Science Monitor*.

[9] Sun Y., Hou Y., Lv N. (2019). Circulating lncRNA BC030099 increases in preeclampsia patients. *Molecular Therapy-Nucleic Acids*.

[10] Gao J., Xu K., Liu H. (2018). Impact of the gut microbiota on intestinal immunity mediated by tryptophan metabolism. *Frontiers in cellular and infection microbiology*.

[11] Chang Y., Chen Y., Zhou Q. (2020). Short-chain fatty acids accompanying changes in the gut microbiome contribute to the development of hypertension in patients with preeclampsia. *Clinical Science (London, England)*.

[12] Wang J., Gu X., Yang J., Wei Y., Zhao Y. (2019). Gut microbiota dysbiosis and increased plasma LPS and TMAO levels in patients with preeclampsia. *Frontiers in Cellular and Infection Microbiology*.

[13] Huang J., Qian Y., Cheng Q., Yang J., Ding H., Jia R. (2020). Overexpression of long noncoding RNA Uc. 187 induces preeclampsia-like symptoms in pregnancy rats. *American Journal of Hypertension*.

[14] Chen F. R., Zheng L. M., Wu D. C., Gong H. M., Cen H., Chen W. C. (2020). Regulatory relationship between lncRNA KCNQ1OT1 and miR-146a-3p in preeclampsia. *Zhonghua Fu Chan Ke Za Zhi*.

[15] Zhang Y., Liu W., Wu M. (2021). PFKFB3 regulates lipopolysaccharide-induced excessive inflammation and cellular dysfunction in HTR-8/Svneo cells: implications for the role of PFKFB3 in preeclampsia. *Placenta*.

[16] Liu J., Lv S. S., Fu Z. Y., Hou L. L. (2018). Baicalein enhances migration and invasion of extravillous trophoblasts via activation of the NF-*κ*B pathway. *Medical Science Monitor*.

[17] Rana S., Burke S. D., Karumanchi S. A. (2022). Imbalances in circulating angiogenic factors in the pathophysiology of preeclampsia and related disorders. *American journal of obstetrics and gynecology*.

[18] Liu J., Yang H., Yin Z. (2017). Remodeling of the gut microbiota and structural shifts in preeclampsia patients in South China. *European journal of clinical microbiology & infectious diseases: official publication of the European Society of Clinical Microbiology*.

[19] Sohn K., Underwood M. A. (2017). Prenatal and postnatal administration of prebiotics and probiotics. *Seminars in fetal & neonatal medicine*.

[20] Ishimwe J. A. (2021). Maternal microbiome in preeclampsia pathophysiology and implications on offspring health. *Physiological Reports*.

[21] Altemani F., Barrett H. L., Gomez-Arango L. (2021). Pregnant women who develop preeclampsia have lower abundance of the butyrate- producer _Coprococcus_ in their gut microbiota. *Pregnancy hypertension*.

[22] Chen X., Li P., Liu M. (2020). Gut dysbiosis induces the development of pre-eclampsia through bacterial translocation. *Gut*.

[23] Lv L. J., Li S. H., Li S. C. (2019). Early-onset preeclampsia is associated with gut microbial alterations in antepartum and postpartum women. *Frontiers in cellular and infection microbiology*.

[24] Paraskevopoulou M. D., Hatzigeorgiou A. G. (2016). Analyzing MiRNA–LncRNA interactions. *Methods in molecular biology*.

[25] Qian X., Zhao J., Yeung P. Y., Zhang Q. C., Kwok C. K. (2019). Revealing lncRNA structures and interactions by sequencing-based approaches. *Trends in biochemical sciences*.

[26] Schoeler M., Caesar R. (2019). Dietary lipids, gut microbiota and lipid metabolism. *Reviews in endocrine & metabolic disorders*.

[27] Wang J., Su Z., Lu S. (2018). LncRNA HOXA-AS2 and its molecular mechanisms in human cancer. *Clinica Chimica Acta*.

[28] Zhang Y., Chen X. (2020). lnc RNA FOXD2-AS1 affects trophoblast cell proliferation, invasion and migration through targeting mi RNA. *Zygote*.

[29] Chen J., Khalil R. A. (2017). Matrix metalloproteinases in normal pregnancy and preeclampsia. *Progress in molecular biology and translational science*.

[30] Cheng D., Jiang S., Chen J., Li J., Ao L., Zhang Y. (2019). The increased lncRNA MIR503HG in preeclampsia modulated trophoblast cell proliferation, invasion, and migration via regulating matrix metalloproteinases and NF-*κ*B signaling. *Disease markers*.

[31] Zhu H., Kong L. (2019). LncRNA CRNDE regulates trophoblast cell proliferation, invasion, and migration via modulating miR-1277. *American Journal of Translational Research*.

[32] Zhou F., Sun Y., Chi Z., Gao Q., Wang H. (2020). Long noncoding RNA SNHG12 promotes the proliferation, migration, and invasion of trophoblast cells by regulating the epithelial-mesenchymal transition and cell cycle. *The Journal of International Medical Research*.

[33] Haugen F., Drevon C. A. (2007). Activation of nuclear factor-*κ*B by high molecular weight and globular adiponectin. *Endocrinology*.

[34] Sha H., Ma Y., Tong Y., Zhao J., Qin F. (2020). Apocynin inhibits placental TLR4/NF-*κ*B signaling pathway and ameliorates preeclampsia-like symptoms in rats. *Pregnancy hypertension*.

[35] Yin A., Chen Q., Zhong M., Jia B. (2021). MicroRNA-138 improves LPS-induced trophoblast dysfunction through targeting RELA and NF-*κ*B signaling. *Cell cycle*.

[36] Vaughan J. E., Walsh S. W. (2012). Activation of NF-*κ*B in placentas of women with preeclampsia. *Hypertension in pregnancy*.

